# Iliopubic rami morphology and its vascular relationships in percutaneous retrograde fixation

**DOI:** 10.1007/s00276-025-03711-y

**Published:** 2025-09-08

**Authors:** Miguel Loureiro Fernandes, Diogo Gonçalves dos Santos, Cristina Costa-Santos, Pedro A. Pereira, André Rodrigues Pinho, Maria João Leite

**Affiliations:** 1 Unit of Anatomy, Department of Biomedicine, Faculty of Medicine, University of Porto, Alameda Prof. Hernâni Monteiro, 4200-319 Porto, Portugal; 2 Orthopaedics and Traumathology Department, ULS São João, Porto, Portugal; 3 Department of Community Medicine, Information and Health Decision Sciences, Faculty of Medicine, RISE-Health, University of Porto, Porto, Portugal; 4 NeuroGen Research Group, Center for Health Technology and Services Research (CINTESIS), Rua Dr. Plácido da Costa, 4200-450 Porto, Portugal; 5 CINTESIS@RISE, Faculty of Medicine, University of Porto, Alameda Prof. Hernâni Monteiro, 4200-319 Porto, Portugal

**Keywords:** Iliopubic rami anatomy, Neurovascular structures, Percutaneous retrograde fixation, Safety iliopubic corridor

## Abstract

**Purpose:**

Pelvic ring fractures involving the iliopubic rami can cause functional impairment. Percutaneous retrograde fixation is a less invasive procedure when compared to traditional open approaches, however precise anatomical knowledge is crucial for safe screw placement. This study aims to describe the morphology of the iliopubic rami, define a safety corridor for percutaneous screw fixation, specially focusing on the relationships between the iliopubic rami and neurovascular structures.

**Methods:**

A retrospective cross-sectional study was conducted on 29 patients using high-resolution computed tomography scans. Measurements included rami shape, narrowest diameter, and distances between the iliopubic rami and neurovascular structures. Statistical comparisons were performed using the Wilcoxon, Mann-Whitney, and Kruskal-Wallis tests.

**Results:**

Triangular and trapezoidal rami shapes were most common being the median narrowest diameter larger in males (8.67 mm) than females (6.83 mm) (*p* = 0.011). The obturator neurovascular bundle was approximately 3 mm from the iliopubic rami, while the external iliac vein and artery were about 5 mm and 11 mm away, respectively. Women also had a greater external iliac vein proximity to the iliopubic rami on the left, compared to men (*p* = 0.032).

**Conclusion:**

Therefore, preoperative imaging is essential to reduce neurovascular risks and given anatomical variations, screw selection should be sex-specific, with 6.5 mm screws for males and 4.5 mm for females. The polygonal rami shapes allow the use of straight plates. The obturator neurovascular bundle is highly vulnerable during screw placement as so it is the external iliac vein compared to the homonym artery, especially in women.

## Introduction

The iliopubic (IP) rami contribute for the formation of the anterior pelvic arch, essential in maintaining pelvic and trunk stability, allowing locomotion, and providing critical attachment sites for significant muscle groups [8, 23].

Pelvic ring fractures represent between 0.3% and 6% of all fractures, but are much more common in polytrauma patients [6], frequently involving the IP rami [16] being predominantly observed in geriatric populations, either derived to low-energy trauma or osteoporosis [15]. Conversely, in younger individuals, high-energy traumatic incidents are the primary cause [10, 15, 29]. Traditionally, open reduction and internal fixation (ORIF) has been employed in managing these injuries, despite its high invasiveness and associated complications [29]. This complexity prompted a shift towards an intramedullary approach, proposed by Lambotte in 1913 [7, 14], and later advanced by Tile, reviewed in [7] but most extensively described by Routt et al. [18, 19]. This technique reduces ORIF-related complications, is simpler to execute, preserves natural bony anatomy, minimizes blood loss and pain, shortens surgery duration, reduces infection rates, and facilitates earlier mobilization [7, 11] while providing stability [24]. It is also used in cases of block-type tumoral resections in the pelvic ring zone in oncologic surgery [1, 3] where fixation of both iliopubic regions is required.

Recent studies on an Arabic cohort revealed that morphological variations of the IP rami impact implant selection for managing pelvic and acetabular fractures [11]. Recognizing this, we consider the retrograde percutaneous fixation technique, also because of its lower fixation rates compared to the anterior approach [24], worthy of continued investigation. The IP rami region has a close proximity to critical neurovascular structures, including the obturator neurovascular bundle and the external iliac vessels [12] and Thampi and collaborators verified the existence of anatomical variations that may predispose to complications [26]. Accordingly, precise anatomical study and a comprehensive understanding of global anatomical and radiological relationships are essential to minimize vascular injury risk during surgery [25]. In this setting, fluoroscopic-assisted computer navigation has been introduced to enhance procedural precision [30].

This study aims primarily to analyze the local neurovascular anatomy, specifically relevant distances to the external iliac vessels and obturator neurovascular bundle when performing percutaneous screw fixation for IP rami fractures. Additionally, it intends to outline the local surgical anatomy to assist in selecting the most appropriate implants for fracture fixation and to contribute to developing safe passageway guidelines for percutaneous rami fixation.

## Materials and methods

### Study design

This study design was a cross-sectional retrospective analysis, level 3 of evidence, in which we aimed to describe the morphology and anatomy of the superior pubic ramus, contributing to determining a possible security hallway for percutaneous retrograde fixation. While defining safety, we were not only concerned about the rami bony limits but mainly its relation to major neurovascular references such as obturator neurovascular bundle and external iliac vessels in a Caucasian population.

The study protocol was approved by the Institutional Review Board – Ethics Committee CHUSJ (CE-393-2024).

## Sample group

The inclusion criteria were: (1) 18 years old or older, (2) Caucasian race, (3) adequate computer topographies (CTs) in dorsal decubitus without fracture or with reduced fracture without displacement, and (4) clear visualization of obturator neurovascular bundle and external iliac vessels. CTs with prosthetic material were also included if they fulfilled the previous criteria and had clear bone limits.

The exclusion criteria were: (1) major fractures with complete distortion of pelvic girdle anatomy, (2) CTs without fracture reduction or highly comminuted fractures, (3) metabolic bone diseases, or (4) primary or secondary tumoral bone diseases.

To fulfill our objectives, we used a convenience sample. We began randomly collecting data from 50 consecutive CTs (100 IP rami) from patients with suspected pelvic fractures evaluated at the emergency department of a Level 1 Trauma Center from 2007 until 2023. However, 52 of them didn’t fulfill the inclusion criteria (3) and were comprised in excluding criteria (1) and (2). Excluding criteria (3) and (4) didn’t exclude a single ramus.

Therefore, our sample includes 29 cases, aged between 18 and 90 (median 50), representing 48 IP rami (25 on the left and 23 on the right). All included patients were Portuguese.

## Morphometric parameters assessment

We used Sectra Uniview ^®^ for our measurements. For bone-related aspects, we used the frequency “Bone”. We confirmed the vessels’ limits in each case in the “Abdominal /Mediastinum” frequency. When all crucial points were marked, we switched to the “Bone” frequency to collect our data. In addition, to increase data measurement quality, we simultaneously used coronal, horizontal, and sagittal views with a localizer tool, allowing us to see each point simultaneously in those plans so that we were certain about the position of our markings.

**Fig. 1:**
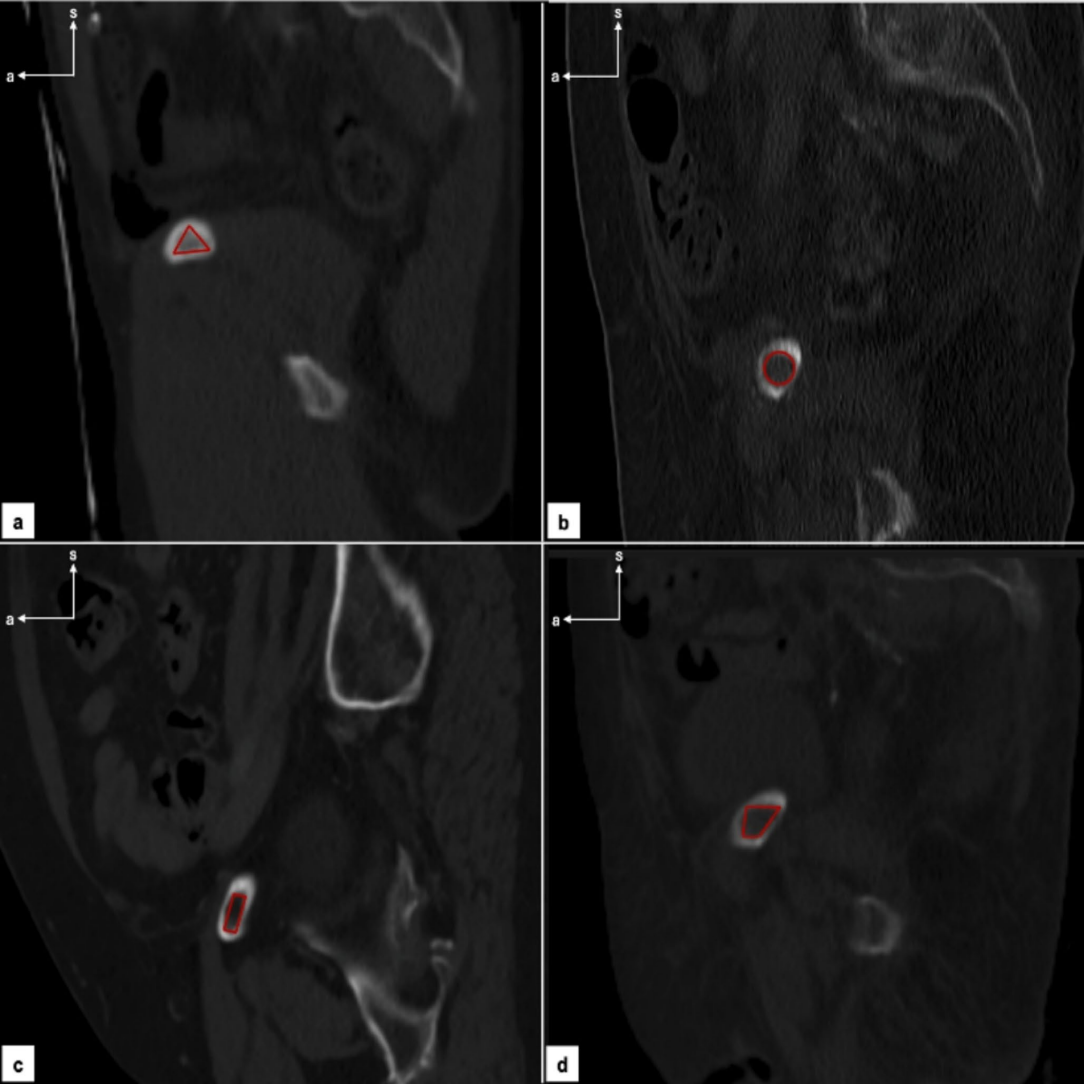
CTs iliopubic rami categories assessment. **a** Triangular; **b** Circular; **c** Quadrangular;** d** Trapezoid. a- anterior; s- superior.

After collecting patient demographic data, we collected the rami shape and its narrowest point diameter. Regarding the shape, we defined 4 categories (a-triangular; b-circular; c-quadrangular; d-trapezoid) (Fig. 1a, b, c and d).

**Fig. 2:**
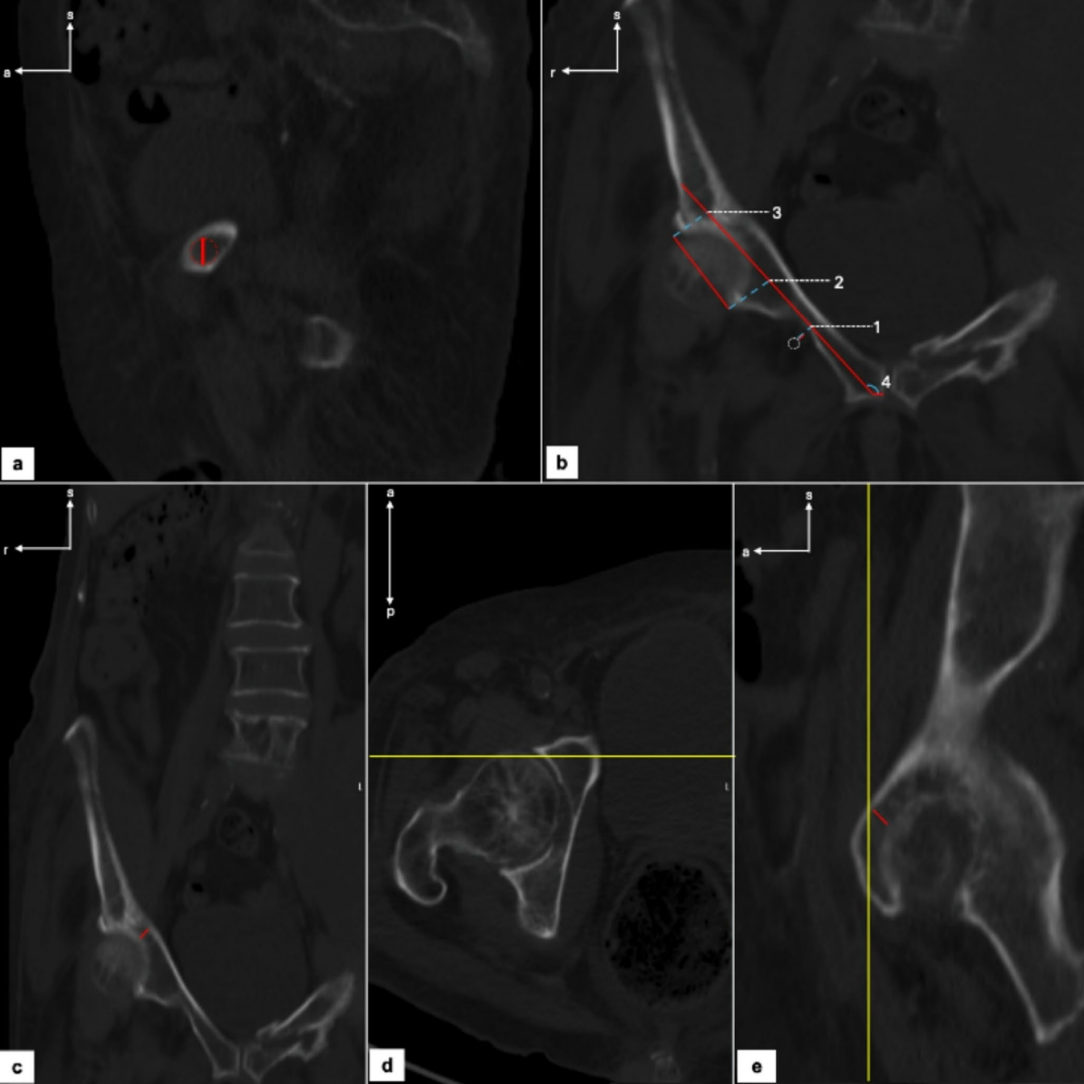
CTs morphometric and neurovascular obturator bundle parameters assessment **a** diameter of IP rami; **b** IP rami axis, neurovascular obturator bundle (white dashed circle) distance to IP rami, distances from the PS to the, neurovascular obturator bundle (1), medial and lateral limits of acetabulum (2) (3) and cephalo-caudal angle (4); **c** minimal coronal acetabulum thickness; **d** predicted screw trajectory in acetabulum; **e** minimal sagittal acetabulum thickness. s- Superior; s- Anterior; r- Right; The yellow line represents the localizer

Regarding the diameter, it was identified by consecutive measures using the largest possible fitting circle within the narrowest zone of the internal diameter of the cortical bone walls in a sagittal view (Fig. 2a).

Secondly, we aligned the tree planes with the help of the localizer to get the most medial point of the pubic symphysis (PS). We could see this as the localizer would pass at the most anterior point of the PS in a horizontal and sagittal plane. Then we would assess the better coronal plane where we were sure to be in the PS and have an optimal view of the osseous pathway by aligning the coronal frame with the horizontal one where we could see the supraacetabular portion where the screw would pass. Afterward, the axis was drawn in a coronal plane, parallel to and respecting the bone limits, until it reached the cortical bone of the iliac bone wing (Fig. 2b). Then we could measure the cephalo-caudal angle of the predicted trajectory of the screw by subtracting 90 degrees from the obtained horizontal angle. We aimed to eliminate potential misleading angles, as some CT scans may not capture the patient in the correct position, potentially distorting the true angle.

For assessing distances related to the neurovascular obturator bundle we identified it and then drew a line from them until the intersection with the screw-predicted axis to assess the distance from the PS to them. However, for the distance to the IP rami we measured it by a perpendicular axis until it reached cortical bone (Fig. 2b). Regarding the acetabulum, we chose 2 points: one in the medial and the other in the lateral extremity to cover all its extension. After that, we also intersected these reference points with the screw-predicted axis (Fig. 2b).

The minimal thickness of the acetabular wall was measured in both coronal and sagittal planes by consecutive measures in the same coronal frame as the measures previously described (Fig. 2c). To measure it, we aligned both coronal and sagittal planes with the horizontal one where we could see the bony portion of the acetabulum where the screw would pass and looked for the minimal thickness possible (Fig. 2d and e). Thereafter, by marking the minimal distance possible we obtained our data.

**Fig. 3:**
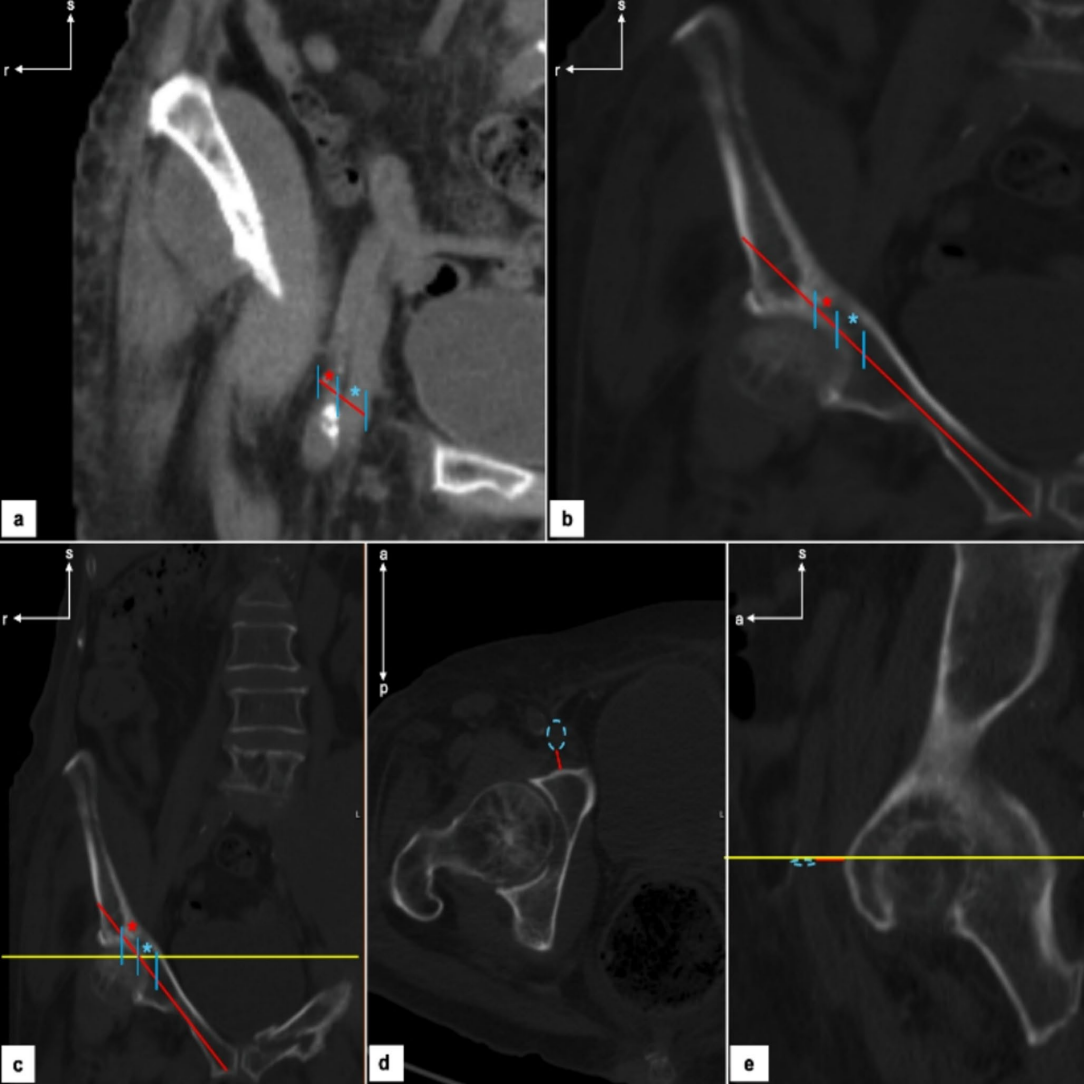
CTs external iliac vessels parameters assessment. **a** identification of external iliac vessels; **b** position of external iliac vessels in relation to IP rami axis; **c** external iliac vein relation to IP rami axis; **d** external iliac vein horizontal distance to IP rami; **e** external iliac vein sagittal (blue dashed circle) distance to IP rami. Red and blue asterisks represent the external iliac artery and vein, respectively. s- Superior; a- Anterior; r- Right; The yellow line represents the localizer

The last measurement group was related to the external iliac vessels. Using the same axis orientation of the screw, we would search the CTs coronal frame where the vessels would cross it, we measured them (with a parallel axis to the original one) (Fig. 3a and b) and then transfer them to the original coronal frame, and proceed as we did with the measures for the distances to the PS. Concerning the vessels’ distance to the IP rami we measured both in sagittal and coronal planes to also assess if they were concordant (Fig. 3c, d and e).

Each measurement was independently taken three times by two different observers, and the median was considered. In the event of a discrepancy, a third researcher (MJA/ARP) was consulted for resolution. The values of the linear measurements are expressed in millimeters (mm).

Regarding Statistical analysis, we assessed the reliability among the three measurements with the intraclass correlation coefficient (ICC). Each measurement was described using the median of the mean values and Inter-Quartil Range (Q1-Q3).

The comparison between left and right sides, for those cases that had both measurements, was evaluated with the Wilcoxon test. The differences in measurements among male and female cases were evaluated for both the left and right sides and compared using the Mann-Whitney test. Cases with reduced fractures (RF), with non-reduced fractures (NRF), and without fractures (non-fractured cases, NF) were evaluated for both the left and right sides and compared using the Kuskal-Wallis test and Mann-Whitney pairwise tests with Bonferroni adjustment. A significance level of 5% was considered.

A post hoc power analysis was conducted to assess the ability to detect group differences in measurements between subgroups of patients with NRF, RF, and NF, separately for the left and right sides. Given the small and unequal sample sizes and the use of non-parametric methods (Kruskal–Wallis test), statistical power was estimated based on conventional effect size benchmarks (Cohen’s f).

## Results

As we explained before, from the initial sample, due to an absence of fulfillment of inclusion criteria or inclusion in the excluding criteria, a total of 29 cases were analyzed with a median age of 50 years (range: 18–90 years), of which 65% were male. Of these cases, 25 had measurements recorded on the left side, with the following distribution of rami shape: triangular in 32%, circular in 12%, quadrangular in 32%, and trapezoidal in 24%. Additionally, 23 cases had measurements recorded on the right side, with ramus shapes distributed as follows: triangular in 30%, circular in 35%, quadrangular in 5%, and trapezoidal in 30%.

All measurements exhibited an ICC greater than 0.9, indicating a high level of reliability among the three repeated measurements.

On the left side, there were 7 cases of NRF, 5 RF, and 13 cases NF. The estimated statistical power for this group was approximately 13% for detecting a small effect size (f = 0.10), 45% for a medium effect size (f = 0.25), and 76% for a large effect size (f = 0.40).

Regarding the post hoc analysis, on the right side, there were 5 cases of NRF, 6 RF, and 12 cases NF. The estimated power was approximately 11% for a small effect (f = 0.10), 40% for a medium effect (f = 0.25), and 73% for a large effect (f = 0.40).

These results indicate that the study is underpowered (< 80%) to detect small to moderate differences among fracture subgroups on either side. Consequently, non-significant results in these comparisons should be interpreted with caution, as they may reflect limited statistical power rather than the absence of true group differences.

**Table 1:** Median and interquartile range of measurements on both the left (*n* = 25) and right (*n* = 23) sides

	Left (*n* = 25) Median (Q1-Q3) mm	Right (*n* = 23) Median (Q1-Q3) mm
IP rami diameter	8.00 (6.83–9.33)	7.67 (6.67–9.67)
*Neurovascular obturator bundle*		
PS- Cortical of the iliac wing	129 (121–135)	126 (121–136)
PS-Neurovascular obturator bundle	39 (37–42)	40 (36–44)
Neurovascular obturator bundle - Cortical of the iliac wing	86 (82–95)	87 (81–95)
Neurovascular obturator bundle - IP rami	3 (2–4)	3 (2–4)
*Acetabulum*		
PS - Begining of acetabulum	65 (58–72)	67 (63–71)
Medial-Lateral limits acetabulum	37 (33–40)	36 (32–39)
Lateral limit of acetabulum - Cortical of the iliac wing	23 (18–31)	23 (19–31)
Acetabulum thickness sagittal	9 (8–11)	10 (8–11)
Acetabulum thickness coronal	10 (8–11)	10 (8–11)
*Angles*		
Horizontal - Axis	142 (139–145)	141 (139–145)
Cephalo-caudal angle	52 (49–55)	51 (49–55)
Axis - Horizontal	38 (35–41)	39 (35–42)
*External Iliac Vessels*		
PS – EIV	67 (63–73)	67 (63–71)
PS – EIA	80 (73–86)	78 (74–82)
IP rami - EIV Horizontal	6 (4–8)	5 (5–8)
IP rami - EIA Horizontal	11 (9–13)	11 (9–15)
IP rami -EIV Sagittal	5 (4–8)	5 (4–8)
IP rami -EIA Sagittal	11 (9–14)	11 (9–15)
PS - Lateral limit of Iliac vessels	88 (82–97)	86 (82–94)
Joint transverse dimensions	21 (19–22)	21 (18–24)
Joint transverse dimensions of the vessels - Horizontal	22 (19–23)	21 (19–23)

Q1: First quartile; Q3: Third quartile; IP: Iliopubic rami; PS: Pubic Symphysis; EIV: External Iliac Vein; EIA- External Iliac Artery;

Table 1 presents the median and interquartile range of measurements on both the left side (*n* = 25) and the right side (*n* = 23).

It was only possible to compare the left and right values ​​in 19 cases (those that had both measurements) and.

we did not find significant differences between the left and right sides in any variable.

**Table 2:** Median and interquartile range of variables in male (*n* = 17) and female (*n* = 8) cases on the left side

	Male (*n* = 17) Median (Q1-Q3) mm	Female (*n* = 8) Median (Q1-Q3) mm	*p*
IP rami diameter	8.67 (8.00-9.67)	6.83 (5.17–7.53)	**0.011**
*Neurovascular obturator bundle*			
PS- Cortical of the iliac wing	129 (120–135)	127 (123–135)	0.793
PS-Neurovascular obturator bundle	39 (38–41)	42 (34–45)	0.381
Neurovascular obturator bundle - Cortical of the iliac wing	86 (82–95)	89 (78–95)	0.560
Neurovascular obturator bundle - IP rami	3 (2–4)	3 (2–4)	0.976
*Acetabulum*			
PS - Begining of acetabulum	63 (56–72)	67 (60–72)	0.560
Medial-Lateral limits acetabulum	38 (34–40)	36 (32–39)	0.308
Lateral limit of acetabulum - Cortical of the iliac wing	22 (19–31)	25 (17–31)	0.838
Acetabulum thickness sagittal	11 (9–11)	8 (8–9)	**0.004**
Acetabulum thickness coronal	11 (9–12)	8 (8–10)	**0.009**
*Angles*			
Horizontal - Axis	142º (138–144)	143º (141–145)	0.641
Cephalo-caudal angle	52º (48–54)	53º (51–55)	0.641
Axis - Horizontal	39º (36–42)	38º (35–39)	0.503
*External Iliac Vessels*			
PS – EIV	66 (63–71)	73 (63–79)	0.162
PS – EIA	78 (74–81)	85 (71–90)	0.503
IP rami - EIV Horizontal	7 (4–9)	3 (1–7)	**0.032**
IP rami - EIA Horizontal	11 (10–13)	10 (7–14)	0.319
IP rami -EIV Sagittal	7 (4–8)	3 (1–6)	**0.013**
IP rami -EIA Sagittal	11 (10–14)	10 (7–14)	0.291
PS - Lateral limit of Iliac vessels	88 (82–92)	94 (80–98)	0.560
Joint transverse dimensions	21 (19–22)	20 (17–22)	0.241
Joint transverse dimensions of the vessels - Horizontal	22 (19–24)	21 (18–22)	0.334

Q1: First quartile; Q3: Third quartile; IP: Iliopubic rami; PS: Pubic Symphysis; EIV: External Iliac Vein; EIA- External Iliac Artery;

**Table 3:** Median and interquartile range of variables in male (*n* = 16) and female (*n* = 7) cases on the right side

	Male (*n* = 16) Median (Q1-Q3) mm	Female (*n* = 7) Median (Q1-Q3) mm	*p*
IP rami diameter	8.67 (7.33–9.77)	6.67 (6.00-9.33)	0.056
*Neurovascular obturator bundle*			
PS- Cortical of the iliac wing	127 (124–138)	120 (117–126)	**0.049**
PS-Neurovascular obturator bundle	40 (37–43)	42 (35–44)	0.973
Neurovascular obturator bundle - Cortical of the iliac wing	88 (85–97)	84 (76–90)	0.061
Neurovascular obturator bundle - IP rami	3 (2–4)	3 (2–3)	0.146
*Acetabulum*			
PS - Begining of acetabulum	67 (63–71)	70 (65–70)	0.815
Medial-Lateral limits acetabulum	36 (32–40)	34 (31–39)	0.403
Lateral limit of acetabulum - Cortical of the iliac wing	27 (19–34)	21 (16–24)	0.109
Acetabulum thickness sagittal	11 (9–12)	8 (7–9)	**0.018**
Acetabulum thickness coronal	11 (9–12)	8 (7–9)	**0.024**
*Angles*			
Horizontal - Axis	141º (139–145)	140º (137–148)	0.815
Cephalo-caudal angle	52º (48–54)	53º (51–55)	0.815
Axis - Horizontal	39º (35–41)	40º (31–43)	0.738
*External Iliac Vessels*			
PS – EIV	66 (64–73)	67 (81 − 71)	0.947
PS – EIA	78 (74–84)	78 (75–82)	0.947
IP rami - EIV Horizontal	6 (5–9)	5 (3–6)	0.171
IP rami - EIA Horizontal	12 (9–17)	10 (7–13)	0.202
IP rami -EIV Sagittal	6 (4–9)	5 (4–6)	0.237
IP rami -EIA Sagittal	12 (9–16)	10 (7–13)	0.180
PS - Lateral limit of Iliac vessels	86 (83–95)	86 (82–91)	0.841
Joint transverse dimensions	20 (18–24)	21 (17–24)	0.920
Joint transverse dimensions of the vessels - Horizontal	21 (19–24)	20 (17–23)	0.402

Q1: First quartile; Q3: Third quartile; IP: Iliopubic rami; PS: Pubic Symphysis; EIV: External Iliac Vein; EIA- External Iliac Artery;

Tables 2 and 3 present the median and interquartile range of variables in male (*n* = 17) and female (*n* = 8) cases on the left side and in male (*n* = 16) and female (*n* = 7) cases on the left and right sides, respectively.

The values in bold in Tables 2 and 3 are those with statistically significant differences.

There were significant differences between males and females in both the sagittal acetabulum thickness (*p* = 0.004 left; *p* = 0.018 right) and coronal acetabulum thickness (*p* = 0.009 left; *p* = 0.024 right) on the right and left sides. Specifically, males exhibited higher median values than females on both sides.

Males also demonstrated higher median values for both the IP rami-external iliac vein (EIV) horizontal distance and the IP rami-EIV sagittal distance compared to females. However, this difference reached statistical significance only on the left side (*p* = 0.032 horizontal; *p* = 0.013 sagittal).

As for the neurovascular obturator bundle, males also have a higher median PS-cortical of iliac wing distance than females, although this difference was only statistically significant on the right side (*p* = 0.049).

**Table 4:** Median and interquartile range of variables in non-reduced fractures (*n* = 7) reduced fractures (*n* = 5) and without fracture (*n* = 13) cases on the left side

	Non-reduced fracture Median (Q1-Q3) mm	Reduced fracture Median (Q1-Q3) mm	Without fracture Median (Q1-Q3) mm	*p*
IP rami diameter	8.00 (7.00-8.33)	9.33 (7.10–9.83)	8.00 (6.33–10.65)	0.590
*Neurovascular obturator bundle*				
PS- Cortical of the iliac wing	129 (120–137)	135 (125–137)	123 (121–130)	0.365
PS-Neurovascular obturator bundle	39 (36–44)	40 (38–41)	39 (34–44)	0.985
Neurovascular obturator bundle - Cortical of the iliac wing	92 (80–99)	94 (87–96)	84 (81–94)	0.477
Neurovascular obturator bundle - IP rami	3 (3–4)	3 (2–6)	2 (2–3)	0.070
*Acetabulum*				
PS - Begining of acetabulum	65 (55–72)	71 (56–73)	63 (61–71)	0.872
Medial-Lateral limits acetabulum	38 (33–39)	39 (34–43)	36 (32 − 29)	0.787
Lateral limit of acetabulum - Cortical of the iliac wing	24 (18–36)	30 (21–32)	22 (18–29)	0.568
Acetabulum thickness sagittal	9 (8–10)	11 (10–12)	9 (8–11)	0.251
Acetabulum thickness coronal	9 (8–11)	11 (10–12)	10 (9–11)	0.237
*Angles*				
Horizontal - Axis	145º (144–148)	142º (137–143)	141º (138–143)	**0.006**
Cephalo-caudal angle	55º (54–58)	52º (47–53)	51º (48–53)	**0.006**
Axis - Horizontal	35º (32–36)	40º (38–43)	39º (37–42)	**0.004**
*External Iliac Vessels*				
PS – EIV	72 (64–80)	67 (66–78)	65 (60–70)	0.171
PS – EIA	85 (72–90)	81 (78–92)	75 (72–82)	0.134
IP rami - EIV Horizontal	7 (4–8)	8 (3–10)	4 (3–8)	0.584
IP rami - EIA Horizontal	9 (8–15)	11 (10–16)	11 (9–13)	0.523
IP rami -EIV Sagittal	7(4–7)	7 (2–10)	4 (3–8)	0.786
IP rami -EIA Sagittal	9 (8–15)	13 (10–17)	11 (9–14)	0.577
PS - Lateral limit of Iliac vessels	98 (82–98)	89 (88–104)	85 (81–91)	0.143
Joint transverse dimensions	21 (18–22)	22 (20–27)	21 (18–22)	0.518
Joint transverse dimensions of the vessels - Horizontal	22 (19–24)	22 (20–26)	20 (18–23)	0.777

Q1: First quartile; Q3: Third quartile; IP: Iliopubic rami; PS: Pubic Symphysis; EIV: External Iliac Vein; EIA- External Iliac Artery;

**Table 5:** Median and interquartile range of variables in non-reduced fractures (*n* = 5) reduced fractures (*n* = 6) and without fracture (*n* = 12) cases on the right side

	Non-reduced fracture Median (Q1-Q3) mm	Reduced fracture Median (Q1-Q3) mm	Without fracture Median (Q1-Q3) mm	*p*
IP rami diameter	7.67 (6.93–10.23)	7.50 (6.24–9.42)	8.87 (6.83–9.67)	0.738
*Neurovascular obturator bundle*				
PS- Cortical of the iliac wing	126 (114–150)	126 (120–129)	126 (122–137)	0.794
PS-Neurovascular obturator bundle	44 (38–46)	42 (38–44)	37 (36–42)	0.180
Neurovascular obturator bundle - Cortical of the iliac wing	80 (74–108)	84 (80–87)	90 (86–95)	0.167
Neurovascular obturator bundle - IP rami	3 (2–4)	3 (2–3)	3 (2–4)	0.624
*Acetabulum*				
PS - Begining of acetabulum	70 (59–72)	67 (60–70)	67 (65–71)	0.717
Medial-Lateral limits acetabulum	34 (33–38)	39 (35–40)	35 (31–39)	0.266
Lateral limit of acetabulum - Cortical of the iliac wing	23 (16–46)	21 (16–26)	26 (21–31)	0.429
Acetabulum thickness sagittal	10 (8–10)	10 (9–10)	10 (7–12)	0.916
Acetabulum thickness coronal	10 (8–14)	10 (8–10)	10 (7–12)	0.830
*Angles*				
Horizontal - Axis	141º (138–146)	141º (139–150)	140º (138–145)	0.639
Cephalo-caudal angle	51º (48–55)	51º (49–60)	50º (48–55)	0.639
Axis - Horizontal	39º (34–42)	39º (30–40)	40º (35–42)	0.660
*External Iliac Vessels*				
PS – EIV	67 (66–78)	67 (61–72)	65 (62–74)	0.426
PS – EIA	80 (75–90)	79 (73–82)	78 (73–85)	0.735
IP rami - EIV Horizontal	5 (4–9)	5 (4–9)	6 (4–9)	0.984
IP rami - EIA Horizontal	12 (9–16)	13 (10–18)	10 (8–15)	0.587
IP rami -EIV Sagittal	5 (4–8)	5 (4–8)	6 (3–8)	0.993
IP rami -EIA Sagittal	12 (9–16)	12 (10–17)	10 (8–14)	0.698
PS - Lateral limit of Iliac vessels	90 (84–103)	87 (80–91)	85 (82–94)	0.602
Joint transverse dimensions	19 (17–26)	19 (17–26)	22 (19–23)	0.883
Joint transverse dimensions of the vessels - Horizontal	19 (17–26)	21 (17–25)	21 (20–23)	0.940

Q1: First quartile; Q3: Third quartile; IP: Iliopubic rami; PS: Pubic Symphysis; EIV: External Iliac Vein; EIA- External Iliac Artery;

Tables 4 and 5 present the median and interquartile range of variables in cases with RF, NRF and NF on the left and right sides, respectively.

**Fig. 4:**
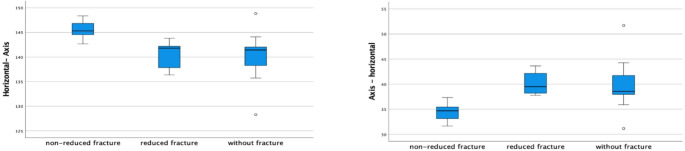
Angles comparison among non-reduced fractures (*n* = 7) reduced fractures (*n* = 5) and without fracture (*n* = 13) cases on the left side

Considering the left side (Table 4) significant differences in horizontal-axis (*p* = 0.006), cephalo-caudal (*p* = 0.006), and axis-horizontal (*p* = 0.004) angles were observed among cases without fracture, with reduced fracture and non-reduced fracture (Fig. 4).

**Fig. 5:**
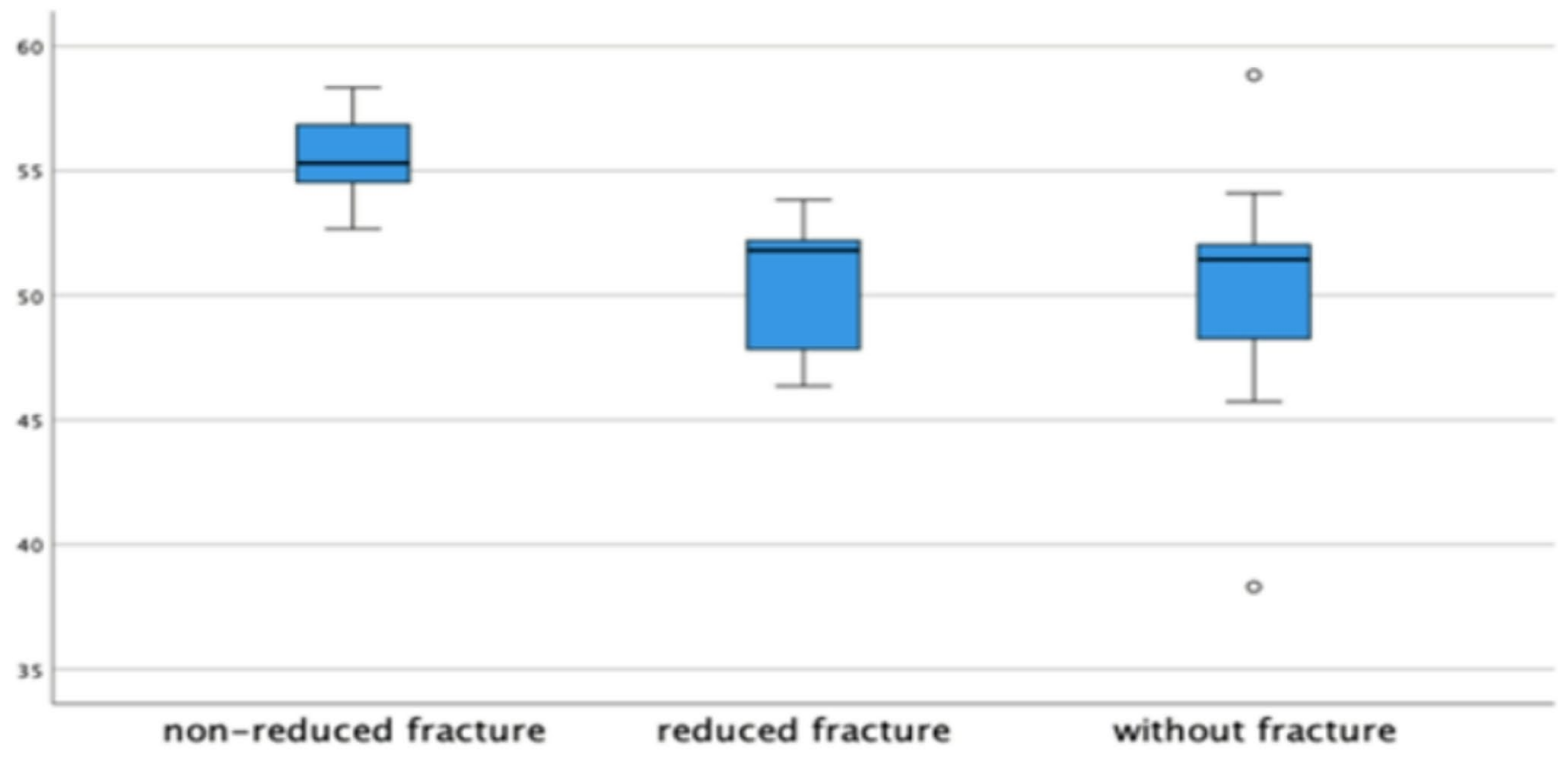
Iliopubic rami diameter comparison among non-reduced fractures (*n* = 7) reduced fractures (*n* = 5) and without fracture (*n* = 13) cases on the left side

For IP rami diameter, there was a significant difference between NRF and RF (*p* = 0.048) as well as between NRF and NF cases (*p* = 0.007). However, no significant difference was found between RF and NF (*p* = 1.000) (Fig. 5).

For the horizontal-axis angle, there was a significant difference between NRF and RF (*p* = 0.048) and NRF and NF cases (*p* = 0.007). However, no significant difference was found between RF and NF (*p* = 1.000).

For the cephalo-caudal angle, there was also a significant difference between NRF and RF (*p* = 0.048) as well as between NRF and NF cases (*p* = 0.007), and no significant difference was found between RF and NF (*p* = 1.000). Similarly, in the axis-horizontal angle, significant differences were identified between NRF and RF (*p* = 0.018) and between NRF and NF cases (*p* = 0.009), with no significant difference between RF and NF (*p* = 1.000).

Considering the right side (Table 5) no significant differences were observed among cases NF, with RF and NRF.

## Discussion

Fluoroscopy allows surgeons to observe screw progression in real-time, reducing, but not eliminating, possible complications [30]. This underscores the importance of anatomical knowledge, including major dimensions and spatial relationships, to further enhance surgical safety. Our study aimed to differentiate itself by focusing with particular interest on the neurovascular relationships of the IP rami, which we consider a key distinction relatively to the existing literature.

### Morphological characteristics

Regarding the IP rami, we found no significant difference between the left and right sides, aligning with expectations, as anatomically, both IP rami in an individual are typically similar. The findings of our study reveal that the most common morphological shapes of the rami are triangular and quadrangular on the left side, whereas circular shapes are more frequently observed on the right. The presence of shapes such as triangular, trapezoidal and quadrangular forms, suggests that adapting a straight plate to the rami or PS would be a viable option when necessary. However, it is important to highlight that the classical description of the morphology of the superior pubic ramus is stated as triangular with three surfaces and borders [23].

The broad age range of participants (18–90 years) and the imbalance in gender distribution present important limitations, as referred in the appropriate section. Age can influence bone quality and morphology, particularly in older adults where osteoporotic changes are more prevalent and larger screws are needed for safe fixation [27]. These factors may affect both the dimensions of the bony corridor and the risk of neurovascular injury during screw placement.

In our sample, the gender differences observed highlight the necessity for careful evaluation and surgical planning, including implant selection. Only adult individuals who had reached local skeletal maturity were included, with cortical bone used as the reference for data collection. Thus, we believe the effect of age on the parameters assessed was minimized.

Without a detailed analysis across different age groups, it remains difficult to fully understand how age-related changes may impact the safety and feasibility of percutaneous fixation in clinical practice. Further studies with age-stratified analyses are needed to better elucidate these effects and to support more tailored surgical approaches.

## IP rami diameter

In our results, we did not find significant differences in IP rami diameter between sides, 8.00 mm (6.83–9.33) on the left and 7.67 mm (6.67–9.67) on the right. However, in a sex sub-analysis, on the left side, median diameters of 8.67 mm (8.00-9.67) in men and 6.83 mm (5.17–7.53) in women were statistically significant, aligning with findings from other studies, as men generally have larger diameters than women. Nevertheless, even though on the right side there are no significant differences, the distance between men 8.67 mm (7.33–9.77) and women 6.67 mm (6.00–9.33) is bigger when compared to the left side. This may be due to the bigger standard deviations on the right side compared to the left. Research performed in a Caucasian population reports median diameters of 9.5 mm in men and 7.5 mm in women [17] and a study on a Chinese population demonstrate 8.76 mm in men and 7.58 mm in women [5], suggesting that these populations are similar with ours.

Despite this, the most used screw sizes are 6.5 mm, 7.0 mm, and 7.3 mm [11], though screws of this size cannot be used safely in most cases. Consequently, studies recommend 6.5 mm screws for men and 4.5 mm for women as generally safer options. Specifically, if we account for a 1–2 mm margin from the cortical bone [25], these screw sizes are suitable for most cases. Larger screws may be considered cases as taller patients or in osteoporotic bone, as each case requires an individualized approach.

## PS – cortical of Iliac wing

We found a median distance of 129 mm in men and 127 mm in women on the left side (*p* = 0.793) and 127 mm in men and 120 mm in women on the right side (*p* = 0.049), only the latter is statistically significant. We used a similar method of measurement as an Arabic study [11] and our right measures are similar to those of the mentioned study. However, regarding both sides’ measurements, when compared to the Japanese (124.6 mm for men and 123.8 mm for women) [25], Chinese (113,83 mm for men and 105,16 mm for women) [5], and Caucasian (110 mm for men and 100.5 mm for women) [17] populations they are bigger, having all of them also not found any statistically significant difference. Nonetheless, Ochs and colleagues (a Caucasian study) [17] used iPlan ^®^ so their measures may be more exact and precise when compared to ours, but if the fractures don’t extend to the acetabulum we don’t need a screw that reaches it [22], so these differences may not have major implications in screw selection in all cases.

### Angles

The software used for the measurements also influenced the cephalo-caudal angle, with our results showing median angles of 52º for men and 53º for women, differing from all other studies. We selected this angle due to its intuitive and easily visualizable nature [17]. An Arabic study reports averages of 49.9º for men and 42.1º for women [11], a Japanese reports 66º and 67º [25], and a Caucasian one reports medians of 42.43º and 42.2º [17], respectively. A Chinese study only provides a mean angle of 42.66º for both sexes [5]. This suggests similar cephalocaudal angles in all populations yet smaller than the Japanese, probably due to regional anatomical variations. Similarly to Japanese [25] and Caucasian [17] studies, we found no significant differences between the sexes.

Comparing with the latter [17], their smaller angles may reflect higher measurement precision. Ochs and collaborators also report that there exist angles in their study, in which the cephalocaudal is comprised, that are independent from the anthropometric parameters sex, body length, body weight and age so we hypotize that some of our differences may not be due to these variables. This discrepancy may also result from our smaller sample size, as discussed in our study’s limitations, or population differences.

The only significant difference between NRF, RF and NF cases was in the cephalo-caudal angles on the left side, which measured 55º for NRF, 52º for RF, and 51º for NF (*p* = 0.006), indicating that proper closed reduction is crucial (Fig. 4). This variation was predicted, as we aimed to exclude from our sample any fractures that were extremely deviated and could significantly distort normal anatomy.

### Supracetabular region

To enhance procedure safety, given that fractures may extend to the supracetabular region, we evaluated its position and thickness. The medial acetabular portion was located at a median distance of 65 mm on the left and 67 mm on the right from the PS, having not found any significant differences between sides or sexes, likely due to our limited sample size.

Regarding thickness, our study found that men had a median of 11 mm in both the sagittal and coronal planes, while women had a median of 8 mm on both sides, a statistically significant difference. An Arabic study reports an average of 8.37 mm for men and 6.45 mm for women [11], whereas a Japanese one found means of 14.2 mm for men and 11.6 mm for women [25], possibly indicating population-based anatomical differences. A Caucasian study presents medians of 11.5 mm for men and 8.5 mm for women [17], aligning closely with our findings which may validate our results. However, these findings are only useful in Nakatani type II and III fractures to have a safe periacetabular fixation and to not damage the hip joint as in Nakatani type I fractures we may not need to approach this region.

### Neurovascular relations

In the CT scans, we could not distinguish the nerve from the vessels, therefore we refer to the obturator canal contents as the “neurovascular obturator bundle.” Due to their close anatomical relationship, an injury in this region could impact both structures.

In our study, the neurovascular obturator bundle is located at a median distance of 39 mm on the left and 40 mm on the right from the PS, with no statistically significant differences found by sex or IP rami condition. We also observed that the bundle was at a median distance of 3 mm from the IP rami on both sides and sexes, regardless of the IP rami’s state, except for the non-fractured IP rami on the left, where a median distance of 2 mm was noted. However, this is not statistically significant (*p* = 0.070).

It is crucial to consider the bundle’s position during screw insertion, as even with the 2 mm safety margin, a 5 mm separation offers limited protection [28]. In cases of cortical violation, these structures can be easily compromised. Additionally, if a “corona mortis” is present, injury to this vessel can be life-threatening due to severe bleeding potential [20, 21]. Although the clinical relevance of this anatomical variation should indeed be highlighted taking into account its prevalence and its surgical implications [20, 21], the evaluation of this parameter was not an aim of our study. A preoperative angio-CT can be valuable to identify the presence of this anastomosis and prevent injury, as each patient’s anatomy varies [4].

Within the above-mentioned canal lies the obturator nerve that if injured may result in sensory loss, pain, and paresthesia extending from the medial side of the thigh to the knee, as well as impaired adduction of the ipsilateral hip joint [9]. If these structures are compromised, it can lead to increased multimorbidity post-surgery and hinder the patient’s full recovery, prolonging rehabilitation. This damage also applies to non-eligible surgical patients, so hemodynamic monitoring and neurologic examination are crucial.

### External Iliac vessels

Regarding the external iliac vessels, their joint transverse dimensions is approximately 21 mm, and are located at a median distance of 67 mm from the PS. The EIV typically lies about 5 mm from the IP rami on both sides, with no significant differences observed. In a sex-based subgroup analysis, we found that the only significant difference was the EIV’s distance to the IP rami on the left side, with women showing shorter distances compared to men, a median of 3 mm versus 7 mm (*p* = 0.013). This difference could be due to our limited sample size, as discussed in our study’s limitations, or may reflect anatomical differences between sexes, as the pelvic ring structure and the position of pelvic organs could contribute to this variation.

This finding aligns with a Japanese study, which reported shorter distances between the IP rami and EIV for women in a plane perpendicular to the screw axis, with means of 5.0 mm for men and 3.9 mm for women [25]. We hypothesize that similar differences may exist on the right side and suggest that a larger sample is needed to confirm these findings.

The EIA is located at a median distance of 80 mm from the PS on the left side and 78 mm on the right side; although no significant differences were noted, it lies 11 mm from the IP rami on both sides. In our subgroup sex analysis, in both sexes coronal and sagittal planes measures were concordant. Men had a median distance from the IP rami of 11 mm on the left and 12 mm on the right, which aligns with a Japanese study’s finding of 12.2 mm in mean, while women had a median distance of 10 mm from both sides, with a Japanese study reporting 10.2 mm in mean [25]. Thus, while we did not find any significant differences, our results are consistent with those reported by the Japanese [25], validating our sample findings. Given that the EIA is a high-pressure vessel, any injury to it may lead to hypovolemic shock and mortality rates ranging from 24 to 60% [2]. Therefore, understanding its precise location may enhance surgical outcomes by providing better protection for this critical structure.

Our data also indicate that the EIV is positioned closer to the bone compared to the EIA, and due to their thinner walls, they are more susceptible to injury in the event of cortical violation, which carries a mortality rate of 55.6% to 77,8% [13].

Regarding these parameters related to the neurovascular structures, additional research with larger sample sizes is essential to further elucidate the neurovascular relationships in this region.

### Clinical impacts

Comprehensive preoperative planning using high-resolution CT is essential to accurately map the iliopubic rami anatomy and adjacent neurovascular structures. Angio-CT plays a crucial role, especially in hemodynamically unstable patients, to identify vascular variants like the corona mortis and mitigate hemorrhagic complications. While angio-CT is preferable for vascular assessment, conventional CT can still effectively inform safe screw trajectories.

The findings underscore the critical importance of precise angular alignment during screw placement, with a recommended safe range close to 50º–55º to minimize the risk of malposition and subsequent neurovascular injury, particularly in displaced fractures.

In terms of hardware selection, the general recommendation favors 6.5 mm screws in males and 4.5 mm screws in females, respecting a 1–2 mm cortical margin, though anatomical variability requires individualized surgical approaches. The proximity of the neurovascular obturator bundle - sometimes as close as 2 mm - and the external iliac vessels highlights the need for meticulous surgical technique to avoid iatrogenic injury. Moreover, the differences observed between male and female anatomy — such as narrower rami in females and closer proximity of the external iliac vein — suggest that surgical planning should incorporate sex-specific anatomical variations.

Additionally, achieving anatomical fracture reduction is paramount for restoring biomechanical stability and optimizing clinical outcomes.

### Limitations

Our study presented several limitations, including a small sample size and the presence of fractures within the study group, which may have affected the generalizability of our findings as described in our post hoc analysis. Additionally, there was a non-uniform distribution of sex among participants, potentially introducing bias and we didn’t evaluate variables as body mass, weight, body length, and age. The study employed virtual trajectories of the screw rather than dedicated software or CTs reconstructions, which may have impacted the precision of trajectory analysis. Furthermore, we did not measure the distances of the constrictions of the IP rami and the acetabulum from the PS, an important parameter for surgical planning. Finally, our measurements were limited to cephalo-caudal angles only, potentially restricting the comprehensiveness of our angular analysis.

## Conclusion

Nowadays, for most cases of IP rami fixation, it is recommended to use 6.5 mm screws in males and 4.5 mm screws in female patients, considering a 1–2 mm margin from the cortical bone. However, due to individual anatomical variations, this should be viewed as a general proposal, evidencing the need for customized surgical strategies. The polygonal rami shapes allow the use of straight plates. The acetabular wall thickness in the Caucasian population, according to our study and Ochs and collaborators [17], present median values of approximately 8–8.5 mm in females and 11–11.5 mm in males, which is crucial for ensuring safe periacetabular fixation in Nakatani type II and III fractures, while less relevant for type I fractures. From a practical perspective, the neurovascular obturator bundle is located at a median distance of 3 mm from the IP rami on both sides and sexes, with as little as 2 mm observed in some cases, in the medial portion of the IP rami, which underscores the need for extreme caution during screw or drill placement to avoid compromising these critical structures. Similarly, the EIV lies approximately 5 mm from the IP rami, and the EIA is positioned at a median distance of 11 mm, both requiring careful consideration during surgical interventions. Furthermore, achieving proper fracture reduction is essential for restoring the original cephalo-caudal angle.

## Data Availability

No datasets were generated or analysed during the current study.
